# Case report: Video-assisted minimally invasive mitral and pulmonary valve replacement as reoperation in patient with situs inversus totalis

**DOI:** 10.3389/fcvm.2023.1053923

**Published:** 2023-08-03

**Authors:** Saad Salamate, Ali El-Sayed Ahmad, Ali Bayram, Sami Sirat, Farhad Bakhtiary

**Affiliations:** ^1^ Department of Cardiac Surgery, University Hospital Bonn, Bonn, Germany; ^2^ Department of Cardiac Surgery, Heart Centre Siegburg, Siegburg, Germany

**Keywords:** minimally invasive surgery, dextrocardia situs inversus totalis, mitral valve, pulmonary valve, reoperation

## Abstract

Dextrocardia with situs inversus totalis is a rare congenital condition. We report herein a first experience of video-assisted minimally invasive mitral and pulmonary valve replacement through right anterior mini-thoracotomy as reoperation in patient with this complex anomaly. The good clinical and cosmetic results demonstrate that this innovative technique can be safely performed even in difficult anatomical conditions.

## Case description

**Figure 1:**
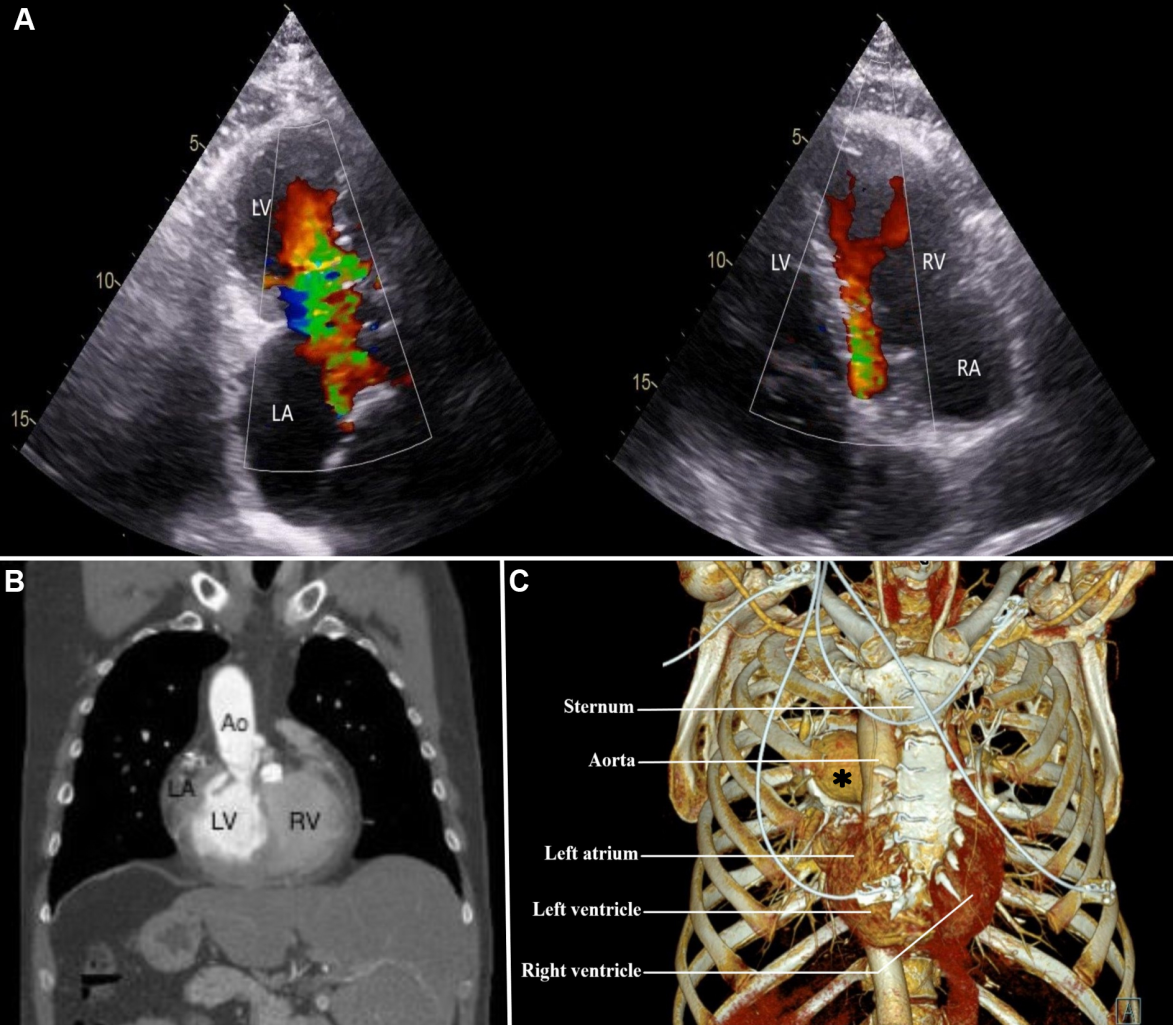
(**A**) preoperative transesophageal echocardiography showing mitral and pulmonary valve regurgitation; (**B**) computed tomography angiography of the chest and upper abdomen, demonstrating a rightward orientation of the left atrium, and liver at the left side; (**C**) computed tomography angiography with 3D reconstruction of the chest, illustrating a right-sided location of the left atrium and ventricle from the sternum. Ao, aorta; LA, left atrium; LV, left ventricle; RA, right atrium; RV, right ventricle. Asterisk, right pulmonary artery.

A 50-year-old man diagnosed with dextrocardia and situs inversus totalis (DSIT) was referred at our institution with symptoms of exertional dyspnea due to severe mitral and pulmonary valve regurgitation for surgical replacement of both valves (Figure 1A). A previous surgical closure of an atrial septal defect was performed through conventional sternotomy 30 years ago. Preoperative computed tomography angiography (CTA) of the chest and abdomen as well as a transthoracic echocardiography (TTE) confirmed the DSIT, citing a light rightward rotation of the apex around the central axis, and showed a very complex anatomy of the heart (Figure 1B). The left atrium (LA) and left ventricle (LV) were placed on the right side, the right atrium (RA) and the right ventricle (RV) on the left side, with the apex lying behind the sternum (Figure 1C). CTA also showed the aorta situated anterior and to right of the main pulmonary artery, with the anatomical right pulmonary artery passing under the aortic arch, suggestive of an anatomically corrected malposition of the great arteries with the I-L-D type (1).

## Surgical technique

**Figure 2:**
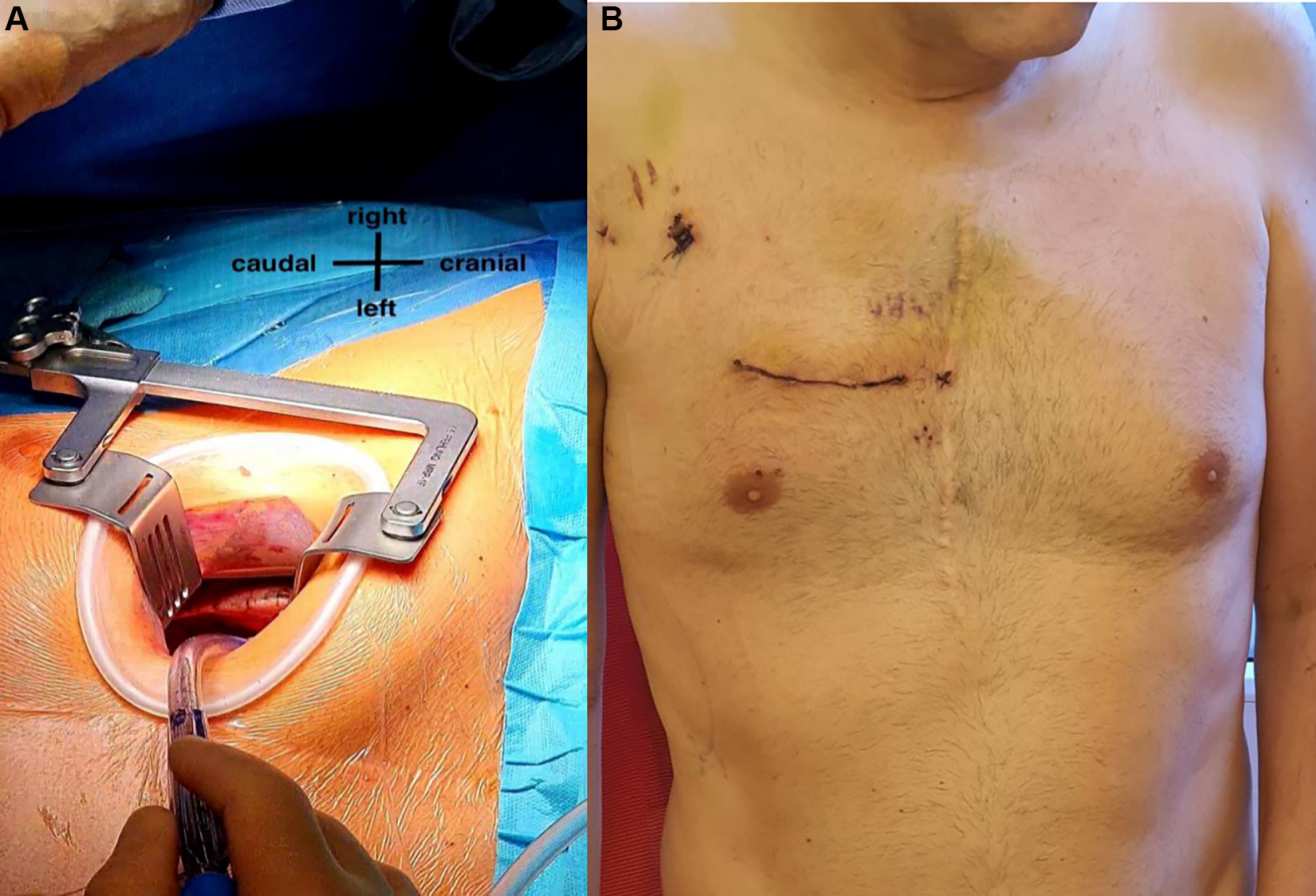
(**A**) right anterior mini-thoracotomy through a 5 cm skin incision at the fourth right intercostal space. (**B**) The patient at 6th postoperative day with the minimal incision right.

After induction of general anesthesia, right vasa femoralis was cannulated through 2 cm skin incision. Cardiopulmonary bypass (CPB) was started. Right anterior mini-thoracotomy through 5 cm skin incision at the fourth right intercostal space (ICS) was performed (Figure 2A). Intercostal tissue has been divided and a soft tissue retractor (Valve Gate™ Soft Tissue Protector, Geister, Germany) has been placed followed by dissection of adhesions until 2 cm above the phrenic nerve to open the pericardium transversely. After exposure of ascending aorta, 2 small incisions for aortic clamp and 3D camera port (Aesculap Einstein Vision, Tuttlingen, Germany) were placed in the third ICS. Cardioplegia catheter (Medtronic DLP 9F, Ref 10012) was inserted into the ascending aorta. The aorta was cross-clamped with Chitwood clamp and crystalloid cardioplegia was administered in an antegrade fashion. Mitral valve (MV) was exposed after dissecting of Waterston’s groove, opening the LA and retracting the anterior LA and septum anteriorly using a retractor (Valve Gate™ Mini-Thoracotomy Retractor, Geister, Germany). The anterior leaflet was totally resected. After annulus sizing, annular sutures were placed and a 29 mm mechanical valve prosthesis (ATS Medical, Inc, Minneapolis, MN) was implanted using automatic fastener technology (Cor-Knot®, LSI Solutions, USA). After closing the LA, pulmonary valve (PV) was exposed through transverse incision of the right pulmonary artery (rPA). After resection of the leaflets and regular decalcification, a sutureless self-expanding biological valve prosthesis size S (Perceval S, Sorin, Saluggia, Italy) was retrogradely implanted, followed by closure of the rPA.

After de-airing the heart, the aorta was declamped and the patient was weaned from CPB after placement of a left ventricular pacing wire. The femoral decannulation was followed by closure of the femoral artery and vein and layered closure of the right groin. A drain trough the cross-clamp incision was placed and the ribs are secured with two FiberWire (Arthrex; Naples, FL, USA). Wounds were then closed in layers. Intraoperative echocardiography showed competent implanted prostheses in the mitral and pulmonary positions.

CPB time and cross clamp time was 108 min and 65 min respectively. Our patient had an uneventful recovery after the surgery. He was extubated on the 1st postoperative day and transferred out of the intensive care unit on the second post-operative day. On the regular station he was ambulated early with good results. On the 4th post-operative day, an elevation of his inflammatory markers entailed the start of empirical anti-biotherapy, which normalized shortly thereafter. The patient was started on oral anticoagulation therapy (Marcumar®) for the mechanical valve. He was discharged home on the 10th postoperative day (Figure 2B). Two days following his discharge, a control TTE was done showing a normal left ventricular ejection fraction and competent pulmonary and mitral valves.

A one-year follow-up revealed that the patient was doing well, with a NYHA I-II classification of dyspnea, and had undergone a cryoablation procedure for atrial fibrillation by his cardiologist. His follow-up TTE also revealed a good left ventricular ejection fraction and good prosthetic valve functions, as well as no paravalvular leak. The patient reported otherwise excellent cosmetic results, and no other pertinent symptomatic changes or clinical findings.

## Discussion

Video assisted minimally invasive approach through RAMT for combined MV and PV surgery as a reoperation represents a considerable technically surgical challenge for cardiac surgeons especially when achieved in a DSIT patient, which is a congenital anomaly with an incidence of 1/10.000 births (2). Due to the complexity of the anatomy in patients with DSIT, the most common access for cardiac surgeries remains the conventional median sternotomy (3, 4). It is a safe and feasible procedure that is associated with good long term outcomes, and allows for the surgical management of a wide spectrum of cardiac diseases with a multitude of anatomical variations, including concomitant cardiac pathologies (5). It is however a considerably invasive approach associated with substantial trauma to the chest wall and intrathoracic structures, and repeat sternotomy is itself associated with early mortality, particularly for adult patients with congenital heart diseases (6).

In contrast, minimally invasive approaches to valvular surgery (MIVS) have already been shown to offer several advantages over classical sternotomy, including a better pain profile, less requirements for blood transfusion, shorter intensive care stay and satisfactory cosmetic results by sparing the surgical trauma of the classical approach (7, 8).

The current literature does not include reports of minimally invasive approaches to valve replacements in patients with situs inversus associated with levocardia and lacks considerably with regards to DSIT, some reports however describe a minimally invasive replacement of isolated aortic valve in patients with DSIT via a left sided access (3, 9).

In patients with situs solitus–normal anatomy, minimally invasive concomitant pulmonary and mitral valve replacement surgery through mini-thoracotomy can be performed from a left-sided approach since the left anterior mini-thoracotomy (LAMT) provides excellent access to the pulmonary valve and right ventricular outflow tract (10).

In DSIT however the anatomy is mirror inverted, which makes a right-sided approach appropriate in order to access the required heart structures providing a good exposure of both the interatrial groove and the right pulmonary arterial tree. Situs inversus can nonetheless be associated with additional anatomical variations and anomalies such as azygos continuation of the inferior vena cava, anomalous pulmonary venous return, or malposition of the great arteries (11). In addition to the challenges of video-assisted minimally invasive concomitant valve surgery as reoperation such as the limited field of view and the steep learning curve, and the adhesions resulting from a previous cardiac surgery by sternotomy, this presents an additional technical burden in the treatment of such pathologies such as in our case, and makes extensive pre-operative investigations and thorough imaging crucial and necessary in order to correctly identify the anatomy of each patient and accordingly plan the best surgical approach.

To the best of our knowledge, we report a first case combining these pathologies and abnormalities with video-assisted minimally invasive concomitant PV and MV surgery from a right sided access. The success in this case was the meticulous preoperative diagnostic evaluation, the recognition of anatomical abnormalities, use of 3D camera and the sutureless self-expanding biological valve prosthesis in the PV offering the patient all the benefits of MIVS.

We accomplished the surgery without particular difficulties and with very acceptable CPB and cross clamp time. One-year Follow-up revealed excellent echocardiographic, cosmetic, and clinical outcomes.

## Conclusion

Video-assisted minimally invasive double valve surgery trough RAMT in a patient with DSIT as reoperation was performed safely with good clinical and cosmetic results.

## Data Availability

The raw data supporting the conclusions of this article will be made available by the authors, without undue reservation.

## Supplementary material

SUPPLEMENTARY VIDEO S1

Video showing step by step the progress of the operation.
